# Conjugated Molecules and Polymers in Secondary Batteries: A Perspective

**DOI:** 10.3390/molecules27020546

**Published:** 2022-01-15

**Authors:** Rudolf Holze

**Affiliations:** 1 Chemnitz University of Technology, Institut für Chemie, D-09107 Chemnitz, Germany; rudolf.holze@chemie.tu-chemnitz.de; 2 Saint Petersburg State University, Institute of Chemistry, 199034 St. Petersburg, Russia; 3 State Key Laboratory of Materials-Oriented Chemical Engineering, School of Energy Science and Engineering, Nanjing Tech University, Nanjing 211816, China

**Keywords:** intrinsically conducting polymers, oligomers, conjugated molecules, secondary batteries, electrochemical energy conversion, electrochemical energy storage

## Abstract

Intrinsically conducting polymers constituting a subclass of macromolecules, as well as a still growing family of large, conjugated molecules, oligomers, and polymers, have attracted research interest for the recent decades. Closely corresponding to the fascination of these materials, combining typical properties of organic polymers and metallic materials, numerous applications have been suggested, explored, and sometimes transferred into products. In electrochemistry, they have been used in various functions beyond the initially proposed and obvious application as active masses in devices for electrochemical energy conversion and storage. This perspective contribution wraps up basic facts that are necessary to understand the behavior and properties of the oligo and polymers and their behavior in electrochemical cells for energy conversion by electrode reactions and associated energy storage. Representative examples are presented and discussed, and an overview of the state of research and development is provided. Particular attention is paid to stability and related aspects of practical importance. Future trends and perspectives are indicated.

## 1. Introduction

**Figure 1:**
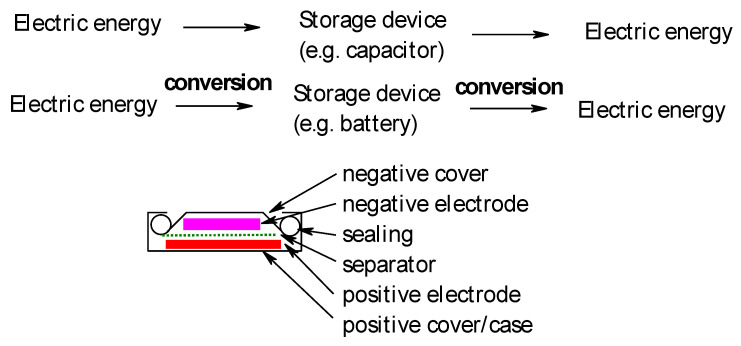
Basic modes of electric energy storage (**top**), cross section of a secondary battery, and coin-type (**bottom**).

In electrochemical devices for energy storage, electric energy is stored by electrochemical transformation (electrode reaction) of electrode materials (active masses) from a state of lower energy (corresponding to a lower value of Gibbs energy or free reaction enthalpy, with respect to the device containing two electrodes; frequently stated data, with respect to only one material or electrode, are meaningless) into a higher state (commonly called the charged state). The ability to store electric energy without such conversion in the electric field of a capacitor or the magnetic field of a coil has become relevant for the first option only recently with the advent of supercapacitors. The second option is relevant only for storage on a small scale for very special applications. Figure 1 illustrates the options schematically.

The associated electrode reactions should proceed fast and reversibly (in its various common meanings: close to equilibrium conditions without excessive losses due to electrode overpotentials caused by slow electrode reactions, in both directions on the same reaction pathway), and they should be possible in both directions as many times as desired by the user without significant deterioration of the participating materials [1,2]. Numerous materials of mostly inorganic origin have been examined, many of which are commonly employed in commercial products [3]. Organic materials have been considered only infrequently, presumably due to concerns about insufficient stability and inferior charge storage capability. An overview of typical data comparing storage capabilities of inorganic and organic materials in Table 1 corrects the latter concerns.

Numbers reported in the representative selection of Table 1 should be considered with care when referring to ICPs. In the case of materials with well-defined charge-storage sites, such as lead ions in PbO_2_ or aluminum atoms in an aluminum electrode in the case of ICPs, charge transfer may proceed at a given location of the polymer chain, but the created charge is delocalized along the molecular chain, leaving the question regarding how many repeat units of delocalization are effective. The answer will provide the oxidation level. At a level equal to 1 all repeat units are oxidized (reduced), but real values tend be much smaller, as discussed below. Such a state may be reached with PANI when the pernigraniline state is reached (for more details see below), but this state is chemically rather unstable and it is commonly considered to be overoxidized, with respect to the application of ICPs presented here. Overoxidation of PANI, as well as other ICPs, has been studied, but as deplored elsewhere [5], no review has been provided so far. It appears safe to state at this point that an increase of storage capability by applying lower/higher electrode potentials will most likely result in faster electrode and battery degradation.

A further comparison of electrode materials, as shown in a representative selection only in Table 1, that must be addressed is ionic and electronic conductivity. Both modes of charge transport depend on concentration and mobility of charge carriers. At first glance, metallic electrodes (e.g., lead in the lead acid battery) appear to be almost ideal. Real electrodes, even of lead or zinc, are porous in order to provide a large surface area. The effective electrical conductance will be decreased. The majority of battery electrode materials—both organic and inorganic—are poor conductors, semiconductors, or almost insulating. Whether the poor electronic conductance of a material is due to low concentration or mobility depends on many factors. In the case of inorganic materials, crystallinity, impurity, and morphology are among the relevant factors. In the case of organic materials, details of molecular structure, degree of polymerization, and molecular ordering can be relevant, and an overview provides more insights [6]. Ionic mobility is relevant when chemical reactions and transport phenomena are connected to the electrochemical redox reaction as the central step in energy conversion and storage. Mobility of lithium atoms and ions in graphite is one of the factors limiting the current generation capability of the negative electrode, and the same applies to intercalation or insertion compounds, serving both as negative or positive electrodes in metal-ion batteries. With organic materials, such connected reactions are less frequent, and in reports on the performance of organic electrode materials, including that presented below, limitations related to ionic mobility are rarely—if at all—addressed. In a wider sense, mobility of ions acting as counter ions for charge balancing may also be relevant. Ions moving in or out of the porous electrode material slower or faster may negatively affect the possible current. Studies explicitly addressing this issue are lacking. Even without exact knowledge of conductivity-related material parameters, electrode design (for a scheme see below) attempts to take care of the related issues more or less intuitively.

Further advantages and limitations of organic materials, in particular ICPs, as battery electrode materials are addressed in detail below.

## 2. The Materials: Intrinsically Conducting Polymers

The term “intrinsically conducting polymer ICP” designates a class of mostly organic oligomeric or polymeric materials, which show electronic conductivity due to mobile charge carriers capable of moving along conjugated segments and hopping between such segments. Materials afforded with electronic conductivity due to the addition of an electronically conducting material, such as graphite powder or metal fibers, and an insulating material are simply and unfortunately, somewhat misleadingly, called conducting polymers, but their other designation as filled polymers is no better and is just as imprecise because it addresses the addition of a second material (forming a composite) that may be insulating and/or may provide some other functionality.

Because of this amazing merger of typical properties of an organic material with those of a metal, i.e., a very inorganic material, ICPs have sometimes been called “synthetic metals”. Unfortunately, the latter term has also been applied to a class of crystalline materials: charge transfer complexes (e.g., N,N’-dicyanonaphthaquinonediimine (DCNNI) and tetrathiafulvalene (TTF). This has resulted in some confusion and has guided frustrated readers to reports on such materials [7,8] when expecting materials for energy storage in ICPs.

**Figure 2:**
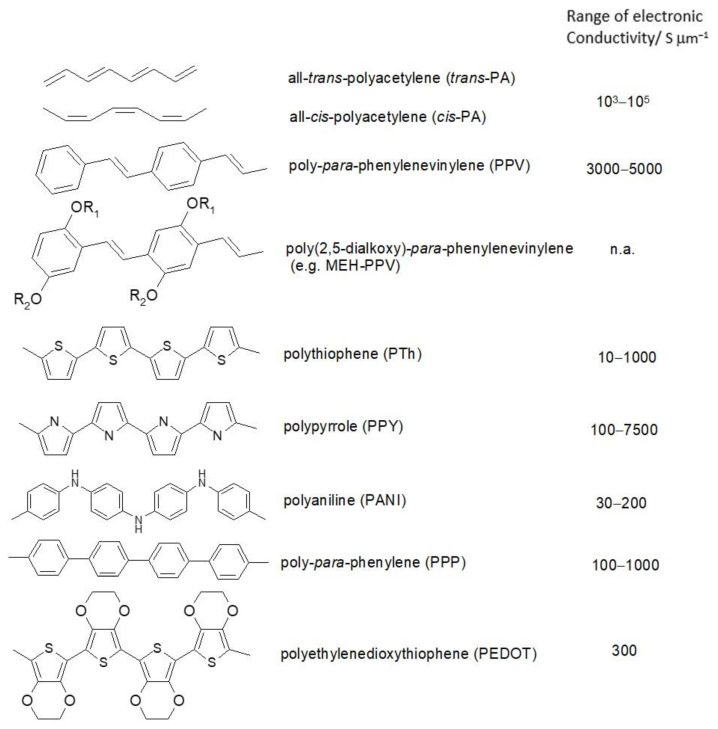
Examples of intrinsically conducting polymers.

ICPs are composed mostly of carbon and hydrogen; sulfur, nitrogen, and oxygen may be present as heteroatoms. A selection of typical examples with simplified molecular structures, systematic names, and generally accepted acronyms and typical values of electronic conductivities is provided in Figure 2. More examples can be found in monographs and handbooks [9,10,11,12,13]. Because of their fascinating combination, organic matter with metal-like properties, ICPs, have attracted tremendous research activities; in particular, the electronic conductivity has been noticed. However, they have actually been known for much longer (for brief historic overviews see [14,15]). Among the numerous applications of these oligo- and polymeric materials showing conjugation already in their repeat units and even more extended conjugation along the molecular chains, their use as active materials in devices for electrochemical energy storage and conversion was suggested many years ago. Initially this meant use in primary and secondary batteries, with the advent of supercapacitors their use in these systems was examined also. The present overview focuses on secondary batteries, but given the ongoing merger of batteries and supercapacitors noticed before [16] and reviewed elsewhere [17], considerations and arguments also apply frequently to supercapacitors; materials may turn up in both types of devices.

For overviews on selected aspects of the application of ICPs in supercapacitors, see [18,19,20,21,22,23,24,25,26]; their use in flexible devices has been discussed in [27,28] and in stretchable devices in [29]. Whether this field of research becomes more enlightened by establishing further terms and acronyms, as in [30], remains an open question.

A different class of polymers with a pronounced electrochemical redox activity is called redox-polymers [13]. In these polymers, localized redox-active functional entities, mostly substituents (or pendant groups) at a molecular backbone, showing neither their own electrochemical activity nor conjugation, enabling electronic charge transport along the backbone, provide an electrochemical response, which might also be of practical relevance for electrochemical energy conversion and storage. If these moieties are attached to the backbone of an ICP, they could possibly disturb conjugation, diminish the ICPs capability to stabilize mobile charge carriers, and thus turn the ICP into a poorly conducting polymer [31]. This can possibly be avoided by substitution with ionogenic moieties showing no redox functionality, such as sulfonate groups [32]; for examples see below. Because they differ chemically from ICPs and because their mode of operation is very different and they are not treated here, reviews are available [33,34,35]. Sometimes, these polymers show electric conductivity enabled by charge transfer between these redox centers by charge hopping. Such polymers are called redox-conducting polymers [36] and are beyond the scope of this contribution.

ICPs can be prepared by a variety of polymerization methods with widely varying features that are more or less suitable for a particular application; for an overview, see [37]. Preparation conditions and experimental parameters can be adjusted by yielding specific morphologies [38]. For more examples and details, see below.

## 3. The Application: Intrinsically Conducting Polymers in Secondary Batteries

Initial optimism regarding real applications of ICPs in numerous fields, including the topic inspected here, has been marred by either observed or at least expected insufficient lack of long-term stability, resulting in electrochemical applications in decreasing storage and current capability. In general terms, this has been addressed for ICPs in secondary batteries in [1] and for supercapacitors in [39]. The sometimes-sloping discharge cell voltages were sometimes claimed as a further drawback. The characteristic property of every ICP, its electronic conductivity, changes as a function of the state of oxidation/reduction of the ICP (e.g., from about 10^7^ S·m^−1^ for doped, oxidized *trans*-PA to 10^−2^ S·m^−1^ for neutral, undoped *trans*-PA). Sometimes (e.g., PANI), protonation plays an additional role (at pH = −0.3 the change covers four orders of magnitude; at pH = 7 only three orders; in organic electrolyte solutions even less). Changes can range across several orders of magnitude. This fascinating property is an unwelcome one in the application discussed here because low conductance tends to limit current capability of an electrode material and increase the Ohmic resistance of electrode material and complete cell. The rather uninspired and fairly traditional approach towards a compensation of this is the addition of conducting materials, such as carbon black or graphite. Unfortunately, this adds dead weight and decreases specific storage capability. Another approach addressed below is the use of thin films on highly conducting substrates or 3D-architectures.

The recent surge in interest in ICPs, i.e., conjugated polymers, for such applications is presumably related to the interest in materials with practically unlimited resources (which is a growing concern with many current battery materials), to the possibility of rather simple handling of used/worn out materials not requiring the specific procedures required for handling heavy metal-containing batteries and devices, to the simple redox reactions not encumbered by intercalation or other possibly slow reaction steps, and to mostly smaller energy usage in preparing these materials under environmentally acceptable conditions (for a broader overview with regard to metal-ion batteries, see [40]). Changes in materials properties can be afforded in most cases using the tools of well-established organic synthesis [31]. Finally, these materials may provide a bridge to the use of renewable materials, such as lignin [41,42,43,44,45,46] or other materials derived from natural resources [47,48,49]. Lastly, these materials may enable or at least simplify construction of flexible and stretchable devices. The pronounced conjugation in ICPs, which in additiin changes as a function of the degree of oxidation/reduction, results in strong interaction between sites where oxidation/reduction proceeds; this is one of the causes of sometimes poorly developed current peaks in CVs [50].

Lacking compatibility with established systems in terms of cell voltage as another barrier initially observed does not appear to be a problem anymore with batteries installed in electronic devices without an option of simply exchanging them, like with standard batteries before. Non-standard cell voltages and other specific features of a given cell thus can be easily integrated into the device. The current level of interchangeability of devices and batteries (with cell voltages of mostly aqueous systems around 1.5 V) is thus lost, but this appears to be no major concern anymore in many cases. Instead, perspectives of all-polymer- or paper-based batteries appear to be more attractive or even realistic [51].

Charge storage in every material proceeds by redox reactions. Another possibility—charge accumulation in the electrochemical double, as employed in electrochemical double layer capacitors EDLC [52,53]—is also of considerable interest but not in the focus of this contribution; it is not related to conjugation in molecules and the associated electronic conductivity. Because an electrode with an ICP as an active mass will establish a double layer with the associated double layer capacity when brought into contact with an electrolyte solution, capacitive contributions to charge storage, with regard to the full cell to energy storage, may be important nonetheless. How to separate these contributions is the subject of an ongoing heated debate [54], and specific aspects with regard to PANI have been reviewed recently [55].

In case of an ICP, charge transfer proceeds via removal (oxidation, most frequently) or addition of an electron (reduction, less frequently observed because of the relatively low electrode potentials needed in most cases and the poor stability of the created species), yielding a radical cation in the former case and a radical anion in the latter case. The radical property is caused by the fact that removal from an electron in a HOMO leaves an unpaired electron, while the addition of an electron into a LUMO also yields an unpaired electron. This radical property has been extensively probed with electron paramagnetic resonance spectroscopy [56]. The term doping, *p*-doping in the case of the oxidation and *n*-doping in the case of a reduction, is frequently used. Different from the quite common meaning in semiconductor physics, where doping means the substitution of atoms (e.g., silicon or germanium) by very small quantities of (dopant) atoms with different valencies (e.g., boron, gallium, ir phosphorus causing a hole or *p*-conduction in the case of the group 3 elements and *n*-conduction in the case of the group 5 element) with ICPs, it apparently refers to the type conduction at first glance: hole conduction upon oxidation; electron conduction in the case of reduction. Whether hole conduction actually means electron movement into the opposite direction is perhaps a more philosophical question in the present context. In any case, the numbers (correct number densities or concentrations) of generated charge carriers is larger by orders of magnitude as compared to semiconductor doping with, e.g., silicon. Adding to the confusion is the further use of dopant ion to designate counter ions moving in or out of the ICP for charge compensation. In this report, neither generation of further confusion nor imprecision are intended and terms related to doping are avoided.

Because of conjugation in the studied oligo or polymer, this electron (or hole) is not located, and the observed EPR-spectra show single lines and no hyperfine structure. This delocalization also provides the electronic conductivity along the molecular chain as a typical feature of an ICP. Movement of charges between molecular chains is provided by electron hopping. Removal of more than one electron is possible; accordingly, there may be no unpaired electrons anymore and the radical property is lost as evidenced again with EPR-spectroscopy [56]. As a consequence of the delocalization of the generated charge(s) that are different from redox processes with metal ions, no well-defined redox potential is created and the electrode potential and consequently the cell voltage change more or less continuously as a function of transferred charged; this is generally called sloping discharge voltage.

**Figure 3:**
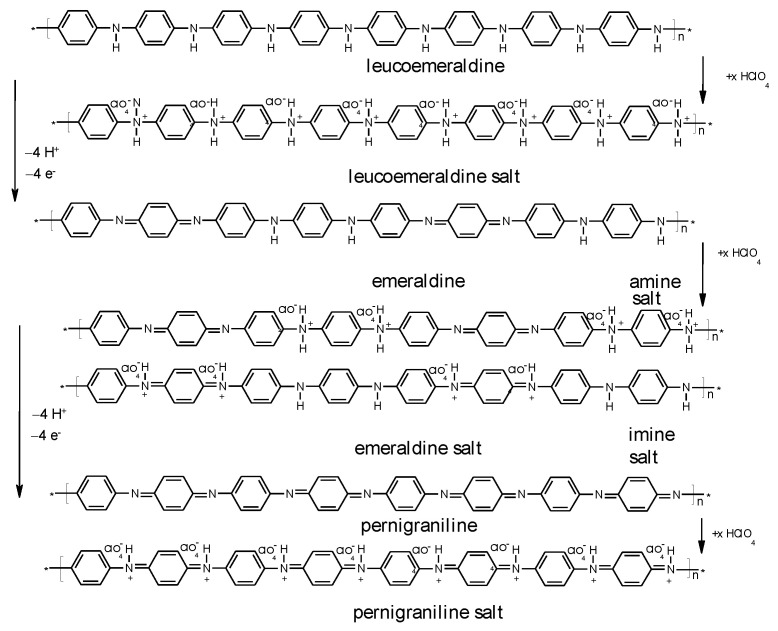
Redox processes of PANI.

These processes can be envisaged with PANI, as shown in Figure 3.

**Figure 4:**
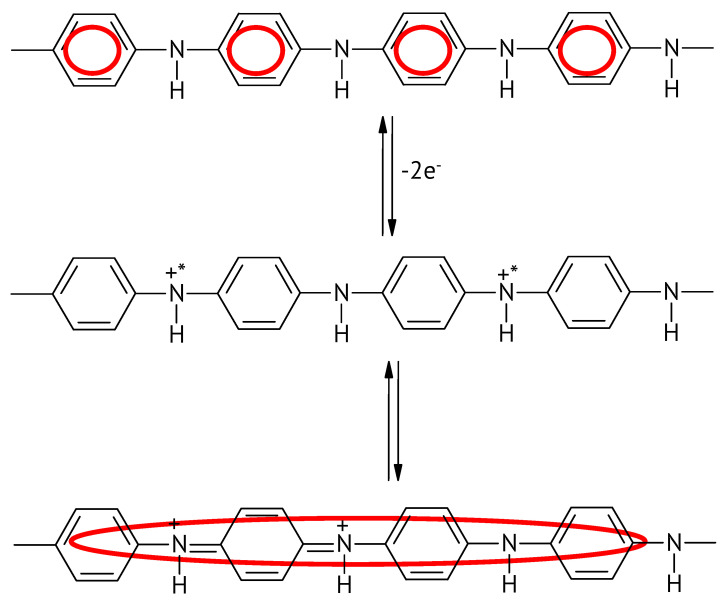
Changing extent of conjugation upon the oxidation of the emeraldine form of PANI.

Upon closer inspection of the oxidation reaction and the associated structural changes of the molecular chain, the changing extent of conjugation, as highlighted in Figure 4, becomes apparent:

The actual extent of conjugation may differ. Determination of molecular weights of ICPs is notoriously difficult; in addition, actual values may differ wildly for a given ICP depending on preparation conditions. This extent should not be confused with the effective conjugation length introduced by Zerbi et al. [57].

**Figure 5:**
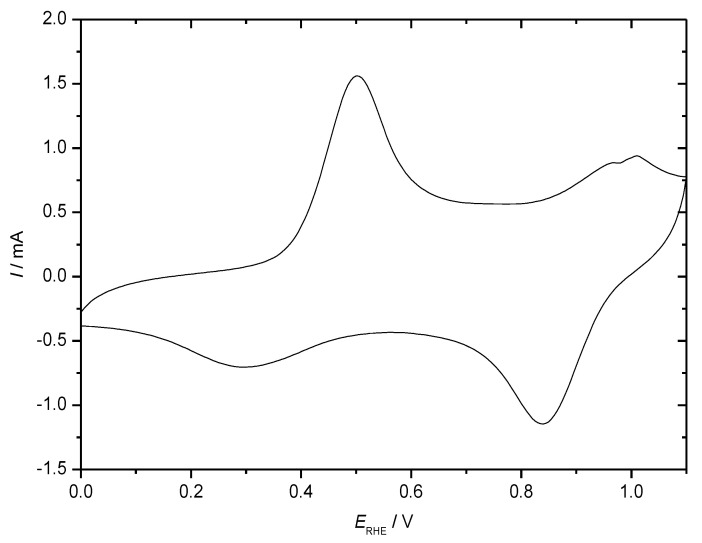
CV of a polyaniline-coated (electropolymerization by 300 electrode potential cycles within the potential range as indicated) stainless steel grid electrode (1 cm^2^) in an aqueous electrolyte solution of 0.1 M aniline + 1 M HClO_4_, d*E*/d*t* = 100 mV·s^−1^, nitrogen purged.

The associated electrochemical response of a film of PANI is shown in Figure 5.

In the positive-going electrode potential scan in a cyclic voltammogram, the first redox transition from leucoemeraldine to emeraldine is observed as the current peak around *E*_RHE_ = 0. 5 V; the second peak indicating the further transition to pernigraniline around *E*_RHE_ = 1 V is not very pronounced because electrode excursion into this region (overoxidation) causes diminished chemical stability of the polymer, resulting in subsequent degradation. In the subsequent negative-going scan, both processes are reversed. The distinctly different peak shapes are due to the different electrochemical and structural situations: When starting at *E*_RHE_ = 0, 0 V PANI is in its poorly conducting state (semi-conducting possibly depending on the definition of this term [6]), providing hardly electronic communication between redox sites, where electron transfer proceeds, this results in a well-developed peak [50]. The second peak recorded with PANI already in its highly conducting emeraldine state is sometimes much broader (as observed here) because of the extensive electronic interactions between redox sites supported by the extended conjugation along the molecular chain. Depending on preparation conditions [58], film thickness, morphology, etc., different results can be observed as shown, e.g., in [59] with a thin film on a gold electrode, providing a well-developed second peak. In the negative going-return scan, the oxidation processes are reversed; the same arguments as made before can be applied to peak shape. In addition to the more or less pronounced current peaks, a rather large residual current between the peaks can be observed. Assignment to a double layer charging current or to a redox process has been the subject of extensive research and discussion; some considerations have been collected in [50]. Because ICPs frequently show an irregular morphology, suggesting a large surface area of the material in contact with the electrolyte solution, considerable values of a double layer capacitance can be expected. This is of particular interest for high-current applications (such as in supercapacitors), where the fast charge/discharge of the double layer sustains much higher currents than the relatively slow redox processes. Because of the relatively fast discharge of EDLC supercapacitors due to dissipation of the accumulated charges in the double layer [60], this contribution is less relevant for secondary batteries considered here. The frequently claimed high fraction of capacitive/pseudocapacitive contributions in battery electrodes is most likely based on a wrong model, as briefly discussed before [54] and pointed out initially elsewhere [61]. Electrochemical impedance measurements may provide an approximation to the desired separation [62,63].

Closely associated with these redox processes are further changes possibly resulting in deterioration of the ICP and in the case of an electrode with an ICP as an active mass in electrode performance.

### 3.1. Shape Change

The redox processes, as shown in Figure 2, are associated with ion movements. For charge balancing upon oxidation, either negative (an)ions have to move into the film or cations (e.g., protons) have to move out. The latter is possible only with polymers that have molecular sites that can be protonated/deprotonated. Whether ion ingress and/or egress proceeds and which process possibly dominates has been studied extensively; for an introduction, see [64]. These processes are associated with volume changes (swelling/shrinking, and shape change) of the polymer because the ions move with some solvation shell. This may negatively affect stability and performance. These changes may result in fragmentation of the polymer, loss of contact between ICP particles, and added conducting carbon in the current collector. Various options to mitigate this shape change have been proposed and examined.

**Figure 6:**
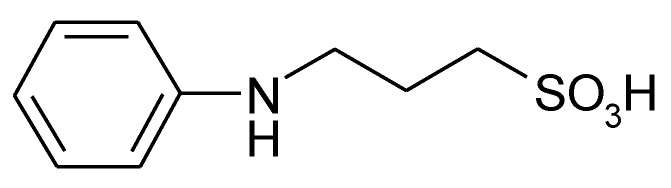
Structural formula of *N*(3-sulpho¬propyl)aniline.

Malinauskas has suggested, in an early review, self-doped ICPs, e.g., self-doped PANI [65]. In such polymers, the presence of fixed negative charges located on anionic groups changes the mechanism of charge compensation during the redox processes of PANI: instead of ingress of anions during oxidation, possibly associated with the problems discussed above, release of cations (e.g., protons or lithium ions) bound at the anionic sites suffices for charge compensation with less detrimental effects. Such self-doped PANI can be obtained in various ways:

- Sulfonation of PANI by chemical post-treatment.
- Polymerization of suitable substituted monomers.
- Copolymerization of aniline and a second suitably substituted comonomer (e.g., copolymerization of aniline and, e.g., *N*-methylaniline and *N*(3-sulphopropyl)aniline [66] (see Figure 6), aniline and *o*-aminobenzene sulfonic acid [67], or *m*-aminobenzoic acid and aniline [68,69]).

A copolymer prepared from aniline and *N*(3-sulphopropyl)aniline (for examples see [66,70]) has been studied. In the former study with *N*-methylaniline as the comonomer, the highest electrochemical activity was observed when a comonomer ratio of 1:1 was established in the electropolymerization solution. This agrees quite well with the degree of doping 0.5 listed above for PANI in Table 1, with half of the repeat units formally participating in the redox reaction. Somewhat surprisingly, this approach has not been exploited in reported research elsewhere. In an investigation employing a copolymer of aniline and *m*-aminobenzoic acid as a positive electrode, 30% capacitance loss after 1000 cycles for a complete supercapacitor cell with a PPy negative electrode was noticed. Unfortunately, no comparison with a similar system without a self-doped ICP was attempted [71]. The copolymer obtained from aniline and metanilic acid by electropolymerization showed significant electrochemical activity in a neutral aqueous electrolyte solution of 0.5 M Na_2_SO_4_, with about 50% capacitance retention when assembled into a symmetric supercapacitor [72].

Because of the pH-sensitivity of a zinc electrode, which is hardly compatible with an acidic aqueous electrolyte solution, preferred for plain PANI, self-doped PANI has been suggested as a form of PANI without the need for an acidic electrolyte solution for a rechargeable zinc-ion PANI battery [73]. As a compromise, a mildly acidic electrolyte solution (pH = 4.5) has been suggested for a PANI/zinc cell [74]. Deposition of PANI as a thin film on an electronically highly conductive 3D-substrate further assisted in ameliorating the problem of moderate electrochemical activity at this pH [75].

The concept of self-doping has rarely been explored beyond the particularly pH-sensitive PANI. An attempt to prepare an oligomeric bis[3,4-ethylenedioxythiophene]3thiophene butyric acid has been reported [76], and its use in “green energy” application has been proposed but not explored.

**Figure 7:**
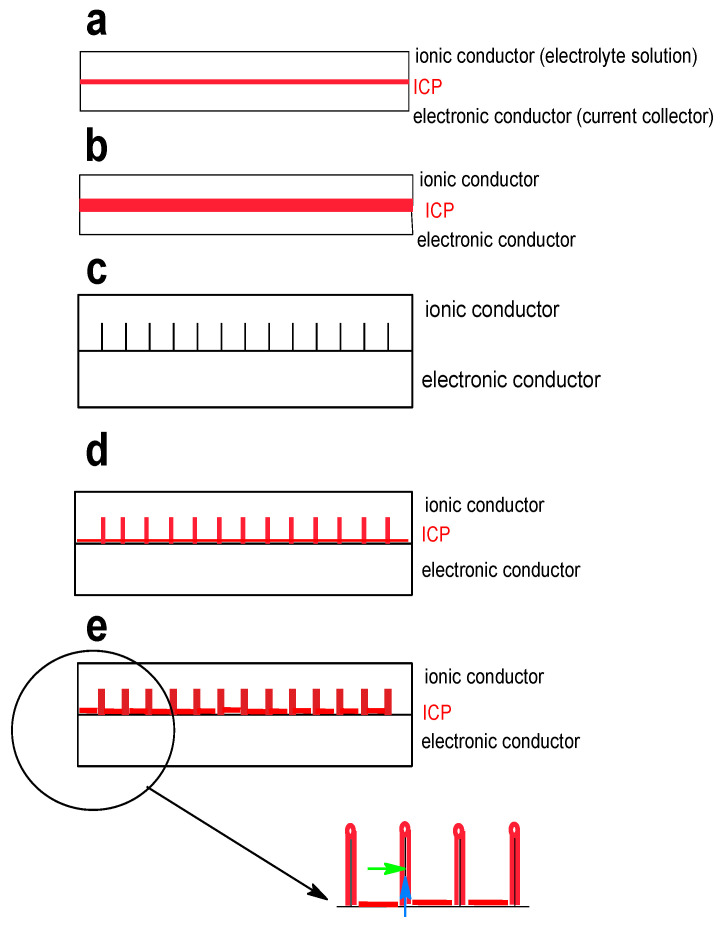
Sketches of battery electrode architectures. Bottom right: black: electronically conducting support, red: active mass with ion (→) and electron (→) pathway).

Another approach examined more frequently is the formation of micro- and nanostructures, allowing shape and volume change to an extent large enough to keep the operation capability of the ICP at an acceptable performance of the material. A comparison of various morphologies (2D: thin film; 3D: microsphere, microtube, microparticles, nanowires networks, nanowire arrays), in particular for supercapacitor applications, has been provided [24]. Nanowire arrays have been suggested as the most promising approach. A similar overview focused on secondary batteries is available [77]. A broader overview focused on 1-D structures of ICPs was provided [78]. In addition to providing more stable electrode structures (morphologies, architectures), nanostructuring may also help to improve performance in terms of current capability. A growing current across the electrode/electrolyte solution interface at a constant interfacial area results in larger deviation of the electrode potential from its equilibrium value, thus a larger overpotential. This unwelcome effect can be limited by increasing the interfacial surface area using a porous electrode or another 3D-structure. Bottom-up strategies, starting with molecular entities assembled in a controlled way into suitable 3D-structures, are available, as well as top-down strategies, starting with bulk materials transformed by milling, evaporation-deposition, etc., into suitable 3D-structures. For ICPs, the former approach is appropriate. Details and examples are presented below. A further concern comes into play: For increased energy of a cell and larger storage capability of an electrode, more material, i.e., a thicker electrode, is required. As illustrated schematically in Figure 7, more mass and current capability limited by Ohmic resistance of the electrode material and the electrolyte solution must be taken into account.

A thin electrode (Figure 7a) may not provide enough mass, a thicker non-porous electrode (Figure 7b) has longer electronic pathways when the electrode reaction still takes place at the ICP/solution interface, an electronically conducting 3D-support (Figure 7c) provides larger interfacial area to be covered subsequently with active mass (Figure 7d), and the actual coating must finally balance electronic and ionic conduction pathways and their respective lengths and contribution to Ohmic resistance (Figure 7e and insert). 3D-supports (instead of smooth metal foils) can be metal grids or meshes, carbon or graphite paper, or carbon structures prepared by pyrolysis of natural materials from biological sources. Further structuring on an even finer level can be afforded by controlled and directed ICP deposition. These considerations also apply to materials and electrodes for supercapacitors, which has been highlighted before [79]. Possible options for various ICPs are addressed below.

Two further modes of ICP and thus electrode deterioration have been noticed and examined in detail.

### 3.2. Peeling Off

Closely related and particularly relevant for electrodes, where the ICP has been directly deposited onto the current collector (i.e., no binder is used), is peeling off from the current collector, leaving the removed particles lost for charge/discharge. Because it is closely related to shape-change, indeed it may be a result of it, remedies against the former may help against peeling off.

### 3.3. Overoxidation

Overoxidation is closely associated with charge/discharge of an ICP in a battery electrode. When the electrode potential of an ICP is moved into a positive direction (see Figure 5), electrooxidation forms cations on the polymer chain. Chemically speaking, they are radicals (polarons is another designation borrowed from solid state physics). These species can be subject to chemical transformation by reaction with electrolyte solution species. When sufficiently positive electrode potentials are reached, additional oxidation of water may yield radical intermediates, which in turn chemically attack and degrade the ICP. The anions moving into the ICP in most cases for charge compensation may affect these reactions, and studies of anion-specific effects show such influences for PANI [80]. Reaction mechanisms and effects of overoxidation vary, and in most studies, decreases in possible electronic conductance, changes of molecular structure, decreases of possible charge storage, and, in the extreme case, complete loss of electrochemical redox response are reported (for examples see [81,82]). Unfortunately, a wider review on this subject seems to be missing; only the degradation of some forms of PEDOT has been inspected more closely in an overview [83]. At first glance, a simplistic view would suggest an easy solution by limiting the potential excursion with suitable electronic circuitry, providing limitation of maximum charge voltage. Unfortunately, practical execution is more complicated. Although the operating voltage limits of batteries have always required appropriate circuit design considerations, the rather low operating voltage of a secondary battery or a supercapacitor with an aqueous electrolyte solution and the sensitivity towards overoxidation require more precise voltage limitation, making the use of batteries and supercapacitors with these materials less attractive. In addition, potential distribution inside of an electrode may leave parts of the material exposed to electrode potentials that are already too high, causing overoxidation. Consequently, other options have been examined (for example, see [24]). Selection of suitable solvents and electrolytes may help to avoid oxidative formation of chemically reactive species (such as hydroxyl radicals when water is used as a solvent).

The redox processes discussed above with PANI, as an example, are one option of charge storage, in addition to double layer storage. The associated ion movement is not related to any redox process at the other electrode, and there is no need for any participating ion to be involved in reactions at both, i.e., the negative and the positive electrode (frequently, the former electrode is called the anode, the latter one the cathode. These terms are correct only during discharge; during charge, the anode turns into a cathode with considerable possibilities of confusion [84]. Consequently, this terminology should be avoided). In case of the very popular metal-ion batteries (e.g., lithium-ion or sodium-ion battery), ions, mostly the metal cations, move between the electrodes. This has caused the nickname “rocking-chair battery”. Their major advantage is the almost completely constant concentration of ions, i.e., cations, in the electrolyte solution, keeping the ionic conductivity and thus the Ohmic internal resistance of the battery constant. Accordingly, the ICP operating in an electrode in such a cell should provide sites for metal ion storage. At this point, the more or less conjugated system of an ICP is not helpful for providing storage sites by itself. Instead, ICPs with attached functional groups have been proposed. An overview is provided in [5], and representative examples are given below.

Reviews and overviews covering various aspects of ICPs, as applied in electrochemical energy conversion and storage, are available [19,31,84,85,86]. Applications of ICPs beyond energy storage by Faradaic reactions as considered here, such as binder or coatings, are included. These reports includes the particularly frequently studied use of ICPs as host material for sulfur in metal-ion sulfur batteries, confining soluble polysulfide species and suppressing the shuttle mechanism. For overviews on this subject, see [5,87].

## 4. Studied Polymers

In this section, some representative ICPs proposed and/or studied for application in secondary batteries are briefly presented. Typical application examples and obtained results are included. For overviews, see [88,89,90].

### 4.1. Polyaniline

Possibly the first secondary battery with a PANI-based positive electrode has been reported in 1990 [91,92]. In the coin-type cell with a nominal cell voltage of 3 V, the negative electrode was prepared by electroplating lithium on aluminum, and the positive electrode was PANI, electropolymerized on a metal mesh, a mixture of propylene carbonate and 1,2-dimethoxyethane with LIBF_4_, and served as an electrolyte solution. Reasons for their rather disappointing marketing have not been reported. It might be speculated that, like with PPy (see below), lacking interchangeability and risks inherent with a metallic lithium electrode in a secondary battery may have been factors.

**Figure 8:**
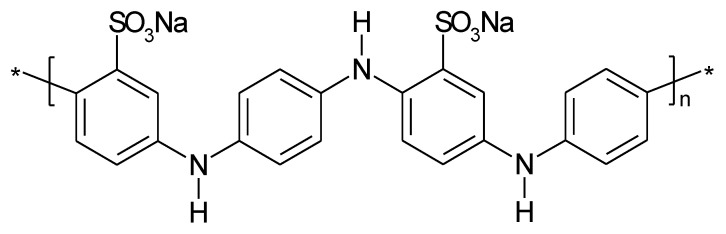
Poly(aniline-co-aminobenzene-sodium-sulfonate).

To improve performance of PANI in a metal-ion battery (e.g., sodium-ion), sulfonated PANI (see Figure 8) has been proposed as sodium storage material [32,93]. As discussed above, this might also be a route to stabilization of the ICP by reducing the effects of shape change associated with counter-ion movement.

Instead of self-doping by suitable substituents, the use of, e.g., PEDOT:PSS (poly(3,4-ethylenedioxythiophene) polystyrene sulfonate) can introduce the same effect: avoiding deprotonation of PANI and loss of electrochemical activity [94]. A nitro-group introduced by chemical copolymerization of aniline and nitroaniline has been proposed as a further option [95].

**Figure 9:**
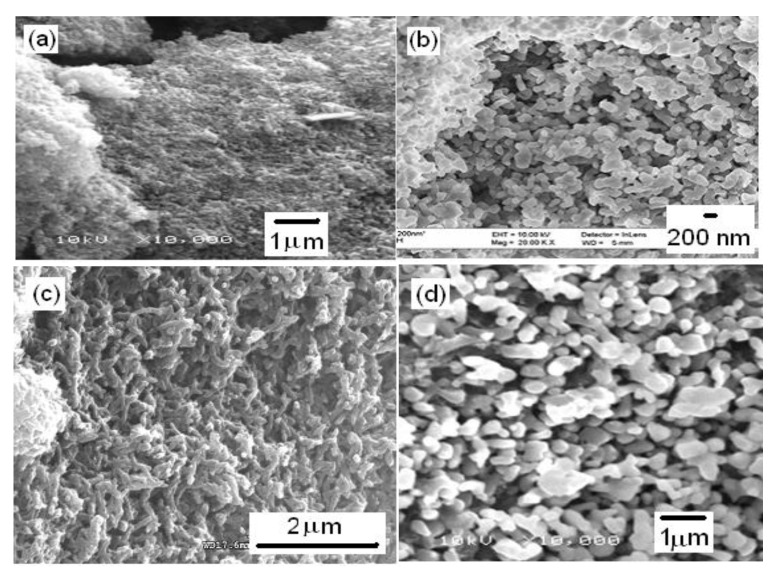
SEM photographs of the nanostructures. (**a**) PANI + oxalic acid (OA), (**b**) PANI + OA + α-alumina, (**c**) PANI + camphoric acid (CA), (**d**) PANI + CA + α-alumina (for further details, see [99]).

Nanostructuring of PANI already during formation by mostly chemical polymerization into, e.g., nanofibers, nanotubes, nanorods, and their arrays on suitable supports (see above) has been addressed. For overviews and examples, see [85,86,96,97,98]. Effects of template materials (both hard and soft) on obtained nanostructures are illustrated in the following microscope images (Figure 9).

PANI and its composites as active mass in electrodes for further metal-ion batteries have been evaluated [100]. Not suffering from the mismatch of suitable pH-values for PANI and a zinc electrode in an aqueous electrolyte solution, addressed in the above combination of PANI as negative electrode with a positive PbO_2_-electrode, yields a type of rocking-chair battery with anions (sulfate) moving between the electrodes [101].

PANI in supercapacitors has been reviewed earlier [102,103]. Further aspects of PANI in electrochemical energy conversion and storage have been discussed before [104].

Vanadium oxygen hydrate intercalated and exfoliated with PANI has been examined as positive electrode material for an aqueous zinc-ion battery [105]. Electronic conjugation of the PANI backbone and the conceivable electrostatic interaction of the conjugated π-system with Zn^2+^-ions and O^2—^-ions of the V-O-layers have been suggested as cause for the growth of the measured effective diffusion coefficient of Zn^2+^-ions by a factor of 10 to 100.

### 4.2. Polypyrrole

**Figure 10:**
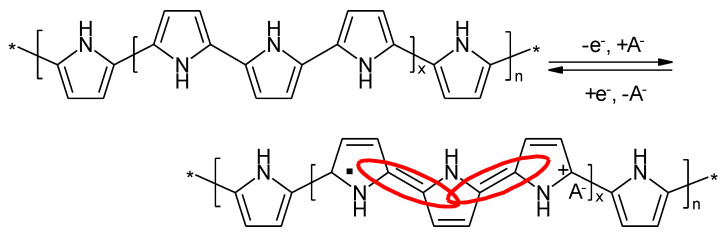
Redox processes of PPy.

The redox processes in a PPy-electrode lack the noticeable pH-dependency observed with PANI in aqueous electrolyte solution, which are schematically shown in Figure 10.

A first cell with two PPy electrode has been described [106]. Although high Columbic efficiencies were noticed, poor charge retention—essential for secondary batteries—was a major drawback, and the high Ohmic resistance of PPy in the neutral state caused a large internal resistance of the cell, a further drawback. The first secondary metal-ion battery with a PPy-film 50 μm thick as a positive electrode, lithium metal foil 100 μm thick, and an electrolyte solution of 0.5 M LiClO_4_ in propylene carbonate was reported in 1987 [107,108,109]. The open-circuit cell voltage when fully charged was 3.6 V, and operational cell voltages between 2.0 and 4 V were recommended. At the latter value, overoxidation of the positive electrode might have become significant; in addition, safety problems related to dendritic lithium deposition may have been one more argument preventing a commercial success of this system. At the time of development, the already mentioned problems of cell voltage incompatibility might have contributed to the commercial failure of this attempt. In a comparative study, PPy as positive electrode with a metallic lithium negative electrode was identified as relatively more stable than PANI and PTh [110]. The same conclusion was reported elsewhere [111].

Polypyrrole nanopipes have been proposed as positive electrode materials for lithium-ion batteries [112]. They combine high-rate capability because of the established structure combined with improved storage capability in comparison to, e.g., activated carbon used for high-rate lithium-ion systems (batteries and capacitors) and mechanical stability.

Electrochemical procedures towards some PPy nanostructures have been developed [113], and further aspects of nanostructures of PPy, as obtained by electrodeposition, have been reviewed elsewhere [114]. PPy nanotubes have shown particularly high electronic conductance [115,116], which in turn is advantageous when they are applied as battery electrode material.

### 4.3. Polythiophene

**Figure 11:**
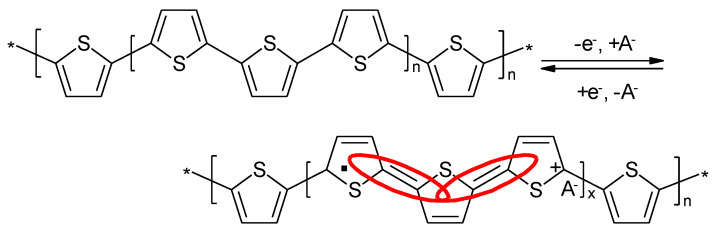
Redox processes of PTh.

First attempts to use electropolymerized PTh as electrode material for secondary batteries have been reported by several groups, starting in 1982 [117,118,119]. The redox process of the oxidation process is schematically shown in Figure 11. The reduction process yielding a radical anion instead of the cation, as shown in Figure 11, is analogous.

**Figure 12:**
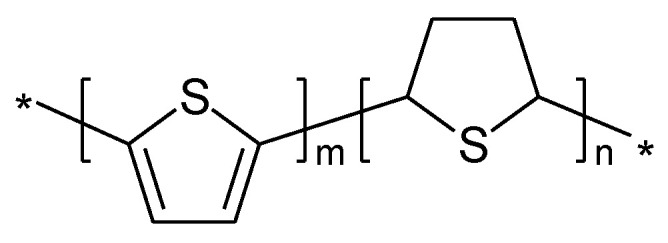
Suggested structure of PTh prepared by chemical polymerization [120].

In case of PTh, the ICPs obtained via electropolymerization (as they were the ones discussed first) and by chemical polymerization, showed striking differences [120]. The authors of the latter report suggested a different polymer sequence (Figure 12).

**Figure 13:**
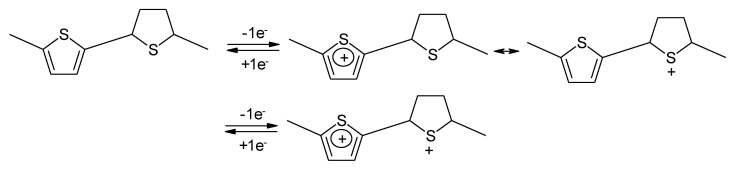
Proposed redox reactions of the PTh illustrated in Figure 12.

Strictly speaking, this polymer is not conjugated. As a consequence, the authors suggest that sulfur-localized redox behavior may also be the possible cause of the less sloping cell voltage. The reaction is schematically illustrated in Figure 13 [120].

**Figure 14:**
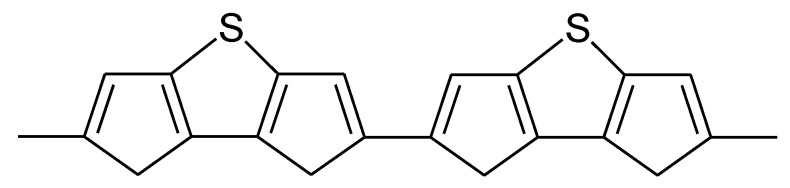
Polydithieno(3,2-b:2′,3′d)thiophene [124,125].

For further details on chemically prepared PTh and its use as a battery electrode, see [121]. An all-PTh battery has been reported [122,123]. Slightly higher doping levels than 0.33 reported for plain PTh (see Table 1) were observed with polydithienothiophene (Figure 14) [124,125,126].

**Figure 15:**
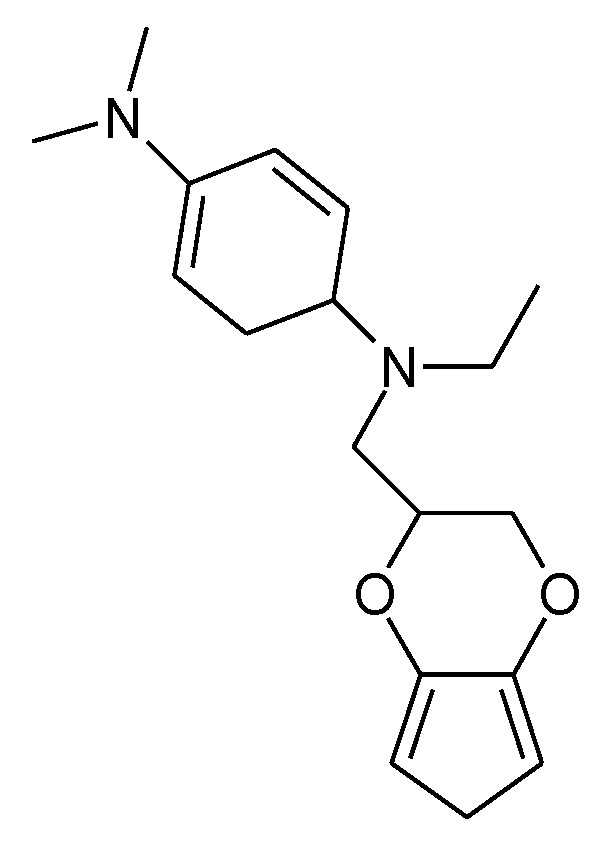
*N*-((2,3-dihydrothieno[3,4-b][1,4]dioxin-2-yl)methyl)-*N*-ethyl-*N*’,*N*’-dimethyl-*p*-phenylenediamine [129].

Closely related to PTh is the ICP PEDOT (see Figure 2). It has been examined with respect to its suitability as an electrode material [127], revealing exceptional stability of this ICP in its oxidized form, even in aqueous electrolyte solutions [128]. More frequently, in electrochemical energy, storage appears to be its use as a binder (for an introduction, see [5]). With further substitution by *N*,*N*,*N*’,*N*’-tetraalkylated-*p*-phenylenediamine (see Figure 15), an ICP showing a charge transfer of up to 2.6 e^-^ per repeat unit could be obtained with electropolymerized material [129]. This corresponds to two redox waves observed in CVs. Possibly the majority of transferred electrons go into the substituent, following Table 1, which shows that a minor fraction might go into the polymer PTh backbone.

### 4.4. Further ICPs

Beyond the three ICPs considered above in detail as representative examples and parent molecules for whole families of substituted molecules (sometimes misleadingly called derivatives), further ICPs have been suggested, and some of them, such as polyindole [130], have been reviewed with respect to their use in secondary batteries. More ICPs and further aspects have been addressed in reports and reviews on the use of ICPs in secondary batteries, etc. [25,131,132,133,134]. Polymers, including ICPs to be used as active masses in metal-ion batteries, have been compared [135]. Conjugation along the polymer backbone has been noted as helpful for increased stability and high-rate capability, presumably because of fast electronic charge transport. Systems with polyacetylene—sometimes considered as the most basic ICP and among the first to be suggested in an all-polymer battery [136]—have been examined. Results appear to go barely beyond academic interest. For an overview, see [84].

## 5. General Aspects and Considerations

**Figure 16:**
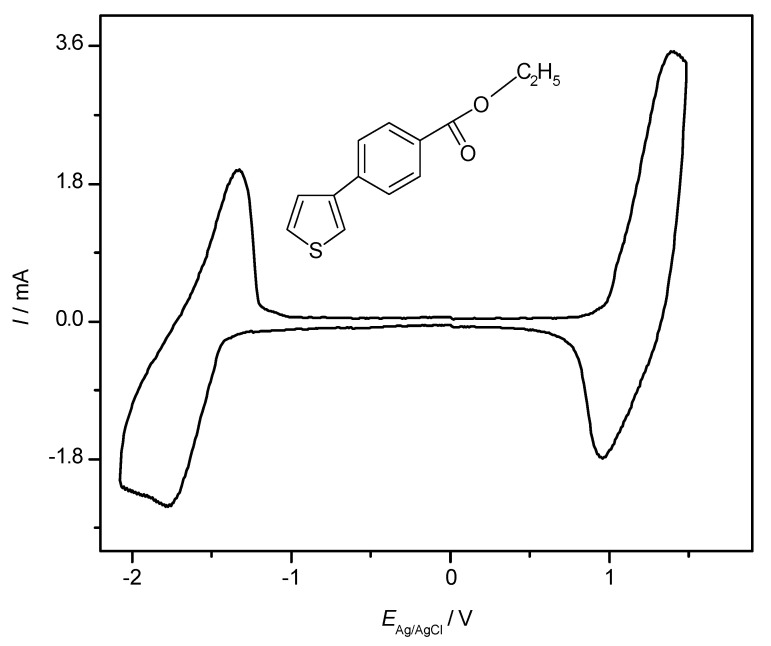
CV of poly(ethyl-*p*-(3-thienyl)-benzoate) coated on a platinum electrode immersed in 0.2 M Et_4_NBF_4_ acetonitrile solution at a scan rate of 100 mV/s. For further details, see [141,142,143,144,145].

With essentially organic polymeric materials as active masses in the electrode(s), an all-polymer battery appears to be feasible. An example of an all-PTh battery has been mentioned above. As pointed out, reduction of an ICP, which would be necessary to generate the charged state of the negative electrode, is of limited practical value when possible at all. Frequently, the reduced state containing radical anions is not sufficiently stable chemically; whether their claimed [31,137] “large impedance” is a further limitation remains unclear. At first glance, a device having the same material in both electrodes with one electrode (the positive one) in the oxidized state and the other one (the negative) in the neutral state (frequently this state is erroneously called the reduced state, possibly because it is created by the reduction of the oxidized state) should provide a cell voltage and be capable of storing energy. Because the electrode potentials will be rather close, even in the charged state, such a cell would not be very attractive, as has been pointed out earlier [138,139,140]. Nevertheless, such arrangements are still pursued, as critically noticed [79]. A second concept employing different ICPs with sufficiently different redox potentials of the neutral-to-oxidized state transition has been considered [138]. In a third attempt, possible only with a few ICPs, mostly from the thiophene family, oxidation and reduction of the same ICP present in both electrodes is used. The CV of a polythiophene prepared by electropolymerization of ethyl-*p*-(3-thienyl)-benzoate (see inset of figure) displayed in Figure 16 shows a typical example.

**Figure 17:**
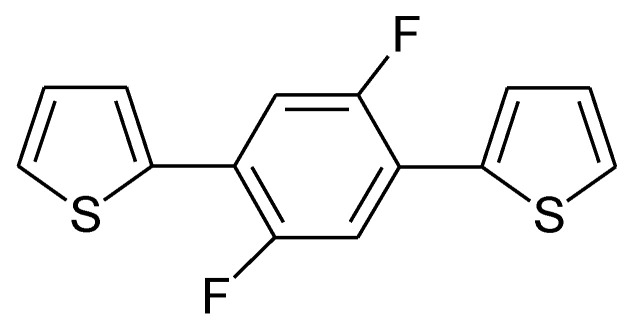
1,4-bis-(2-thienyl)-2,5-difluorophenylene [147].

Reduction of substituted PTys can be further supported by suitable substituents, such as fluorine [146,147,148,149,150] (for an example see Figure 17) or phenyl units in poly(3-phenyl-thiophene) [139]. For further examples, see [31]; for general considerations, see [151].

The term “all-polymer battery” refers to the active masses, except for the electrolyte solution. In an all-solid-state battery, this concept is developed further by inserting a solid electrolyte or at least a gelled-polymer electrolyte instead of the liquid electrolyte solution commonly used. Some concepts beyond the scope of this report have been presented elsewhere [31].

A frequently encountered drawback of many ICPs is their insolubility in most solvents and their generally poor processability. Notable exceptions are some polymers prepared from substituted monomers and oligomers with molecular weights (i.e., chain lengths) that are sufficiently large to show typical properties of an ICP and short enough to enable solubility or at least preparation of stable dispersions, like with PEDOT.

Modeling and theoretical tools have been applied to conjugated oligo and polymers for secondary batteries and to full devices. For an early example, see [152].

Nanostructured ICPs, in particular with nitrogen as heteroatom, have been used as starting materials for the pyrolytic generation of nanostructured carbon as electrode material or as supporting material. The nanostructure of the starting material frequently serves as a template for the obtained carbon material. In addition, the nitrogen heteroatoms support high electronic conductance [112].

## 6. Conclusions and Outlook

The use of intrinsically conducting polymers as active materials in secondary batteries is attractive, with respect to availability of the materials, their environmental compatibility, and their sustainability. Selection of monomers and ICPs should take into account complexity of their synthesis, and ICPs requiring complex synthesis procedures may be attractive from a scientific point of view but may turn out to be economically disappointing. Stability of the materials is still insufficient, or at least disappointing in many cases. Accordingly, there should be more extensive lifetime studies under realistic conditions (in terms of minimum cycle number, depth of discharge, and currents). If possible, actual usage settings (such as electronic supervision circuits suppressing overcharge and overoxidation) should be considered. A deeper understanding of their behavior, in particular with respect to degradation processes based on intensified research, yielding insights into options of enhancing stability by avoiding degrading operating conditions, might help along the way.

## Figures and Tables

**Table 1:** Selected electrochemical data of some electrode materials ^1^.

Material	Molecular Weight of Repeat Unit/g	Oxidation Level */-	Theor. *Q ^#^*	Measur. *Q*/F·g^−1^
PANI	93	0.5	750 F·g^−1^	240
PPy	67	0.33	620 F·g^−1^	530
PTh	84	0.33	485 F·g^−1^	-
PEDOT	142	0.33	210 F·g^−1^	92
Porphyrin C_20_H_14_N_4_	310.35	1	311 As·g^−1^	-
Quinone/HQ	108	2	1787 As·g^−1^	-
Ferrocene	185	1	522 As·g^−1^	-
Li	6.939	1	13,904 As·g^−1^	-
Al	26.98	3	10,728 As·g^−1^	-
PbO_2_	239	2	807 As·g^−1^	-

^1^ Data taken from [4]. PANI = polyaniline, PPy = polypyrrole (also poly (2,5-pyrrolylene)), PTh = Polythiophene (also poly (2,5-thienylene)), PEDOT = poly-3,4-ethylenedioxythiophene. * Oxidation level, also “dopant level”, reports the fraction of oxidized repeat units and the number of electrons transferred in the electrode reaction. ^#^ gravimetric charge density can be stated with respect to the electrode reaction in units As·g^−1^ or in case of a material where no clear electrode reactions can be stated as an amount of charge stored within a change of electrode potential in units of As·g^−1^ (i.e., F·V^−1^·g^−1^).

## Data Availability

Not applicable.
